# Trehalose alleviates high‐temperature stress in *Pleurotus ostreatus* by affecting central carbon metabolism

**DOI:** 10.1186/s12934-021-01572-9

**Published:** 2021-04-07

**Authors:** Zhi-Yu Yan, Meng-Ran Zhao, Chen-Yang Huang, Li-Jiao Zhang, Jin-Xia Zhang

**Affiliations:** 1 Institute of Agricultural Resources and Regional Planning, Chinese Academy of Agricultural Sciences, Beijing, 100081 China; 2 Key Laboratory of Microbial Resources, Ministry of Agriculture and Rural Affairs, Beijing, 100081 China

**Keywords:** Trehalose, *Pleurotus ostreatus*, High‐temperature stress, Glycolysis, Pentose phosphate pathway

## Abstract

**Background:**

Trehalose, an intracellular protective agent reported to mediate defense against many stresses, can alleviate high-temperature-induced damage in *Pleurotus ostreatus*. In this study, the mechanism by which trehalose relieves heat stress was explored by the addition of exogenous trehalose and the use of trehalose-6-phosphate synthase 1 (*tps*1) overexpression transformants.

**Results:**

The results suggested that treatment with exogenous trehalose or overexpression of *tps*1 alleviated the accumulation of lactic acid under heat stress and downregulated the expression of the phosphofructokinase (*pfk*) and pyruvate kinase (*pk*) genes, suggesting an ameliorative effect of trehalose on the enhanced glycolysis in *P. ostreatus* under heat stress. However, the upregulation of hexokinase (*hk*) gene expression by trehalose indicated the involvement of the pentose phosphate pathway (PPP) in heat stress resistance. Moreover, treatment with exogenous trehalose or overexpression of *tps*1 increased the gene expression level and enzymatic activity of glucose-6-phosphate dehydrogenase (*g6pdh*) and increased the production of both the reduced form of nicotinamide adenine dinucleotide phosphate (NADPH) and glutathione (GSH), confirming the effect of trehalose on alleviating oxidative damage by enhancing PPP in *P. ostreatus* under heat stress. Furthermore, treatment with exogenous trehalose or overexpression of *tps*1 ameliorated the decrease in the oxygen consumption rate (OCR) caused by heat stress, suggesting a relationship between trehalose and mitochondrial function under heat stress.

**Conclusions:**

Trehalose alleviates high-temperature stress in *P. ostreatus* by inhibiting glycolysis and stimulating PPP activity. This study may provide further insights into the heat stress defense mechanism of trehalose in edible fungi from the perspective of intracellular metabolism.

**Supplementary Information:**

The online version contains supplementary material available at 10.1186/s12934-021-01572-9.

## Background

*Pleurotus ostreatus* is cultivated worldwide and is widely studied due to its high nutritional, economic, medical, and ecological value [1]. However, cultivation of *P. ostreatus* is still mainly performed in the shed in many developing countries [2], and various abiotic stresses, such as high-temperature, oxidation, osmotic pressure, and acid-base stresses [3], are often encountered during this process. Among these stresses, heat stress is especially serious, and the frequent occurrence of high-temperature spawn-burning has caused heavy economic losses [4]. Therefore, the heat stress response mechanism and high-temperature tolerance of *P. ostreatus* have been important scientific research topics.

Trehalose, a nonreducing disaccharide, is widely distributed in nature [5]. Trehalose is reported to play an important role in tolerance to multiple stresses in numerous species. Trehalose accumulation increases the resistance of *Salmonella enterica* to high salt, low pH, and hydrogen peroxide [6] and increases thermal and osmotic stress resistance in *Listeria monocytogenes* [5]. Trehalose is required for resistance to high ethanol concentrations, heat, and freezing stresses in *Saccharomyces cerevisiae* [7] and to oxidative stress in *Candida albicans* [8]. Moreover, an increase in heat resistance and redox stability is accompanied by an increase in trehalose in the ascospores of *Neosartorya fischeri* [9]. Mutants of the plant pathogen *Ustilago maydis* that do not produce trehalose show increased sensitivity to oxidative, heat, acid, ionic, and osmotic stresses [10]. Trehalose can also ameliorate the radial growth defects in *P. ostreatus* under heat stress [2].

The stress defense mechanism of trehalose has also been studied widely. Most earlier studies attributed the antistress effect of trehalose to its nature as a compatible solute, and currently, the mechanisms of trehalose in stress defense comprise three main aspects. First, trehalose can stabilize the lipid bilayer via the formation of hydrogen bonds between itself and several phospholipid molecules in mycelial fungi [11]. Second, it can act with reactive oxygen species (ROS) to alleviate oxidative damage; specifically, trehalose production can be induced by ROS [2, 12, 13] and increase the total antioxidant capacity to eliminate ROS [14–16]. These properties reveal the complex relationship between trehalose and ROS. Third, trehalose can stimulate selective autophagy in human chondrocytes to ameliorate oxidative stress-mediated mitochondrial dysfunction and endoplasmic reticulum (ER) stress [17]; this finding identifies the stress resistance mechanism of trehalose on a new level and shifts focus to a possible relationship between trehalose and mitochondria.

In addition, intermediate metabolites in trehalose metabolism have attracted our attention. Glucose 6-phosphate (G-6-P), the substrate for trehalose synthesis, is the product of hexokinase (HK) in glycolysis and also the starting metabolite in the pentose phosphate pathway (PPP). Glycolysis, the PPP, and the tricarboxylic acid cycle, the core components of cell metabolism, are collectively referred to as central carbon metabolism [18, 19]. Thus, trehalose metabolism and the central carbon metabolism pathways are closely related. Our recent research has shown that the levels of the intracellular metabolites trehalose and intermediates in the trehalose metabolism pathway undergo significant changes during the response to high-temperature stress. Moreover, we found that central carbon metabolism plays an important role in the response to high-temperature stress [20]. Although the mechanism by which trehalose contributes to heat stress defense in *Pleurotus* has long been studied, few studies have considered this process from a metabolic perspective. We sought to determine whether the mechanism by which trehalose defends against high-temperature stress is related to central carbon metabolism in *Pleurotus* mycelia. Thus, we determined the effect of trehalose on central carbon metabolism by adding exogenous trehalose and using trehalose-6-phosphate synthase 1 (*tps*1) overexpression transformants. The results are anticipated to provide a foundation for research on and the application of trehalose in the heat stress defense of edible fungi.

## Results

### Trehalose accumulates under high‐temperature stress

**Fig. 1:**
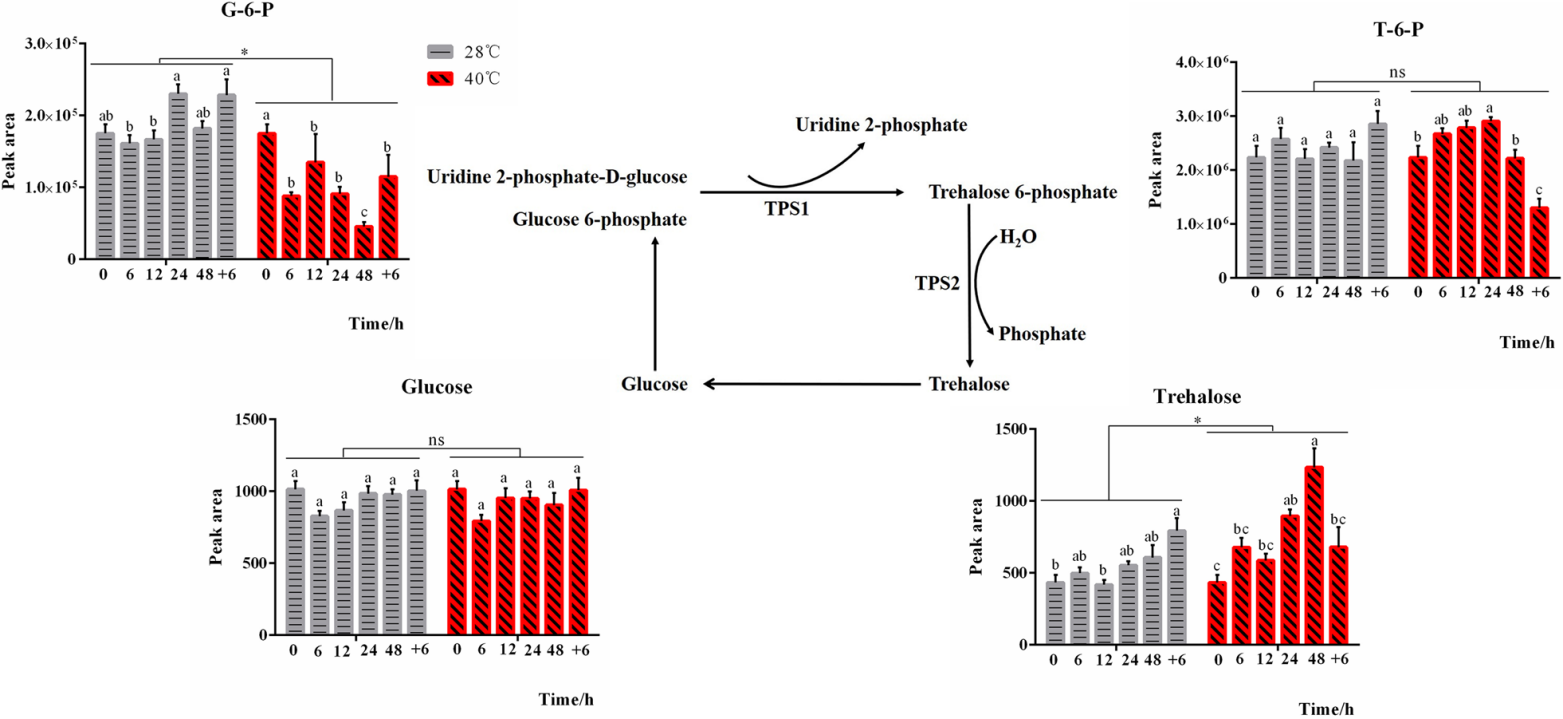
Trehalose accumulates under high-temperature stress. The changes in intracellular metabolites in *P. ostreatus* mycelia treated with heat (40 °C) for 0–48 h were detected by LC–MS, and the intermediate metabolites in trehalose metabolism showed different changes. The changes in the contents of different metabolites under high-temperature stress are indicated in the graph. The abscissa of the graph shows the treatment time, among which 48 + 6 h means treatment with heat for 48 h and then recovery for 6 h at 28 °C. The ordinate of the graph shows normalized peak area data, which represent the relative contents of various metabolites. The data used to construct the graph are the average values of seven biological repeats and are expressed as mean ± SEM values. TPS1: trehalose-6-phosphate synthase 1, TPS2: trehalose-6-phosphate synthase 2, G-6-P: glucose 6-phosphate, T-6-P: trehalose 6-phosphate. The significance analysis was first conducted within 28 °C or 40 °C group separately, and the significant differences were marked with different lowercase letters over the columns (*P* < 0.05 according to the Tukey or Kruskal-Wallis test); and then conducted between 28 and 40 °C whole groups, the significant difference were indicated by *, *P < 0.05 according to the Bonferroni test, ns means no significance. The lowercase letters between 28 and 40 °C groups have no comparability in significance analysis

We performed one LC-MS metabonomic assay on the intracellular metabolites of *P. ostreatus* mycelia under heat stress (40 °C) for 0–48 h and found a significant change in the trehalose content. Trehalose accumulated significantly (550.53 ± 30.33 vs. 751.41 ± 52.58, *P* = 0.001) under high-temperature stress and reached a maximum at 48 h (Fig. 1). In addition, the contents of related intermediates in the trehalose metabolism pathway also changed differentially under high-temperature stress (Fig. 1). G-6-P showed an overall decreasing trend under high-temperature stress (1.91 × 10^5 ^± 7.00 × 10^3^ vs. 1.08 × 10^5 ^± 1.04 × 10^4^, *P* < 0.001). Due to the adequate supply of glucose in the CYM medium, the glucose content did not change significantly (945.46 ± 23.34 vs. 937.53 ± 28.02, *P* > 0.05). Surprisingly, the trehalose 6-phosphate (T-6-P) also did not differ significantly (2.4 × 10^6 ^± 9.4 × 10^4^ vs. 2.4 × 10^6 ^± 1.02 × 10^5^, *P* > 0.05) under high-temperature stress. However, the accumulation of trehalose in *P. ostreatus* mycelia under heat stress may indicate a role for trehalose in the *P. ostreatus* heat stress defense [2].

### **Trehalose can alleviate the increase in glycolysis in*P. ostreatus*under heat stress**

**Fig. 2:**
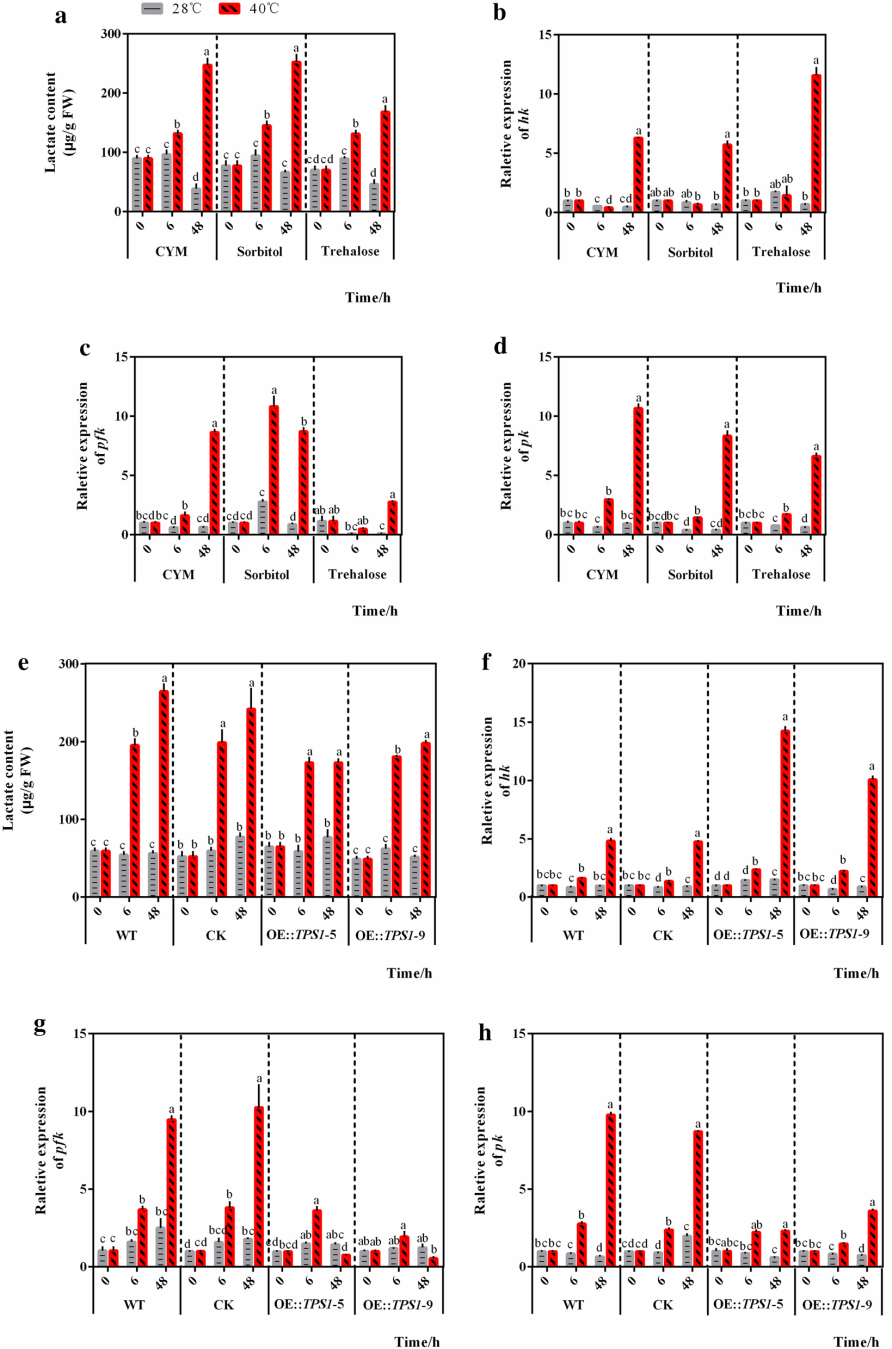
Trehalose can alleviate the increase in glycolysis in *P. ostreatus* under heat stress. The WT strains were transferred to new CYM medium with or without trehalose or sorbitol after 5 d of culture and treated with heat (40 °C). Mycelia were then collected for **a** the lactate content assay and measurement of the relative gene expression levels of **b** *hk*, **c** *pfk*, and **d** *pk*. The WT strain, CK strain, and *tps*1 overexpression strains OE::TPS1-5 and OE::TPS1-9 were treated with heat (40 °C) after culture at 28 °C for 5 d. Mycelia were then collected for determination of **e** the lactate content and relative gene expression levels of **f** *hk*, **g** *pfk*, and **h** *pk*. n = 3; the data are expressed as mean ± SEM values. FW: fresh weight. The significance analysis was conducted within CYM, Sorbitol, or Trehalose group; within WT, CK, OE::TPS1-5, or OE::TPS1-9 group separately, and the significant differences were marked with different lowercase letters over the columns (*P* < 0.05 according to the Tukey or Kruskal-Wallis test), the lowercase letters among different groups have no comparability in significance analysis

The lactic acid that accumulates in mycelia under heat stress can inhibit the growth of *P. ostreatus* [20], but addition of trehalose for 48 h significantly reduced lactic acid accumulation in mycelia under heat stress (Fig. 2a) compared with the CYM (247.63 ± 10.41 vs. 168.46 ± 9.86, *P* = 0.005) or sorbitol (252.66 ± 12.15 vs. 168.46 ± 9.86, *P* = 0.006) controls. This result indicated the relationship between trehalose and lactic acid under heat stress. Since we once found that continuous high temperature stress can accelerate glycolysis [20], and lactic acid comes from the end product of glycolysis [21], we sought to determine whether the addition of trehalose affected glycolysis. To achieve this goal, we measured the transcription levels of genes encoding key glycolytic enzymes containing hexokinase (*hk*, Fig. 2b), phosphofructokinase (*pfk*, Fig. 2c), and pyruvate kinase (*pk*, Fig. 2d) after adding trehalose. As expected, treatment with exogenous trehalose for 48 h significantly downregulated the transcription of *pfk* (8.66 ± 0.20 vs. 2.77 ± 0.06, *P* < 0.001, Fig. 2c) and *pk* (10.67 ± 0.34 vs. 6.63 ± 0.23, *P* = 0.001, Fig. 2d) compared with the CYM control. Surprisingly, exogenous sorbitol could also reduce *pk* expression after 48 h of heat stress, which might indicate a possible association between sorbitol and pyruvate metabolism. However, unlike the trend in *pfk* and *pk* gene expression, *hk* gene expression (Fig. 2b) showed a significant increase in the presence of trehalose for 48 h under heat stress compared with the CYM (6.31 ± 0.04 vs. 11.59 ± 0.63, *P* = 0.001) or sorbitol (5.72 ± 0.28 vs. 11.59 ± 0.63, *P* = 0.001) controls.

The lactate content and gene expression levels of *hk*, *pfk*, and *pk* were measured in the transformants OE::TPS1-5 and OE::TPS1-9. TPS1 catalyzes the first reaction in trehalose synthesis. Overexpression of *tps*1 significantly alleviated the accumulation of lactic acid after 48 h of heat stress (264.44 ± 8.89 vs. 173.06 ± 4.41, *P* = 0.001; 264.44 ± 8.89 vs. 198.18 ± 2.93, *P* = 0.002) (Fig. 2e) and decreased the relative gene expression of *pfk* (9.48 ± 0.22 vs. 0.77 ± 0.01, *P* < 0.001; 9.48 ± 0.22 vs. 0.55 ± 0.09, *P* < 0.001) (Fig. 2 g) and *pk* (9.78 ± 0.14 vs. 2.30 ± 0.07, *P* < 0.001; 9.78 ± 0.14 vs. 3.61 ± 0.08, *P* < 0.001) (Fig. 2 h) in the transformants OE::TPS1-5 and OE::TPS1-9 compared with the WT strain. However, the relative *hk* gene expression was still increased in the OE::TPS1-5 and OE::TPS1-9 transformants after 48 h of heat stress (Fig. 2f). These results indicated that the heat stress-relieving effects of exogenous and endogenous trehalose were similar. In addition, trehalose might alleviate the increase in glycolysis in *P. ostreatus* under heat stress, as shown by the decreased content of lactate and the downregulated expression of genes encoding the key enzymes in glycolysis, with the exception of *hk*.

### **Trehalose can enhance pentose phosphate pathway activity in*P. ostreatus*under heat stress**

**Fig. 3:**
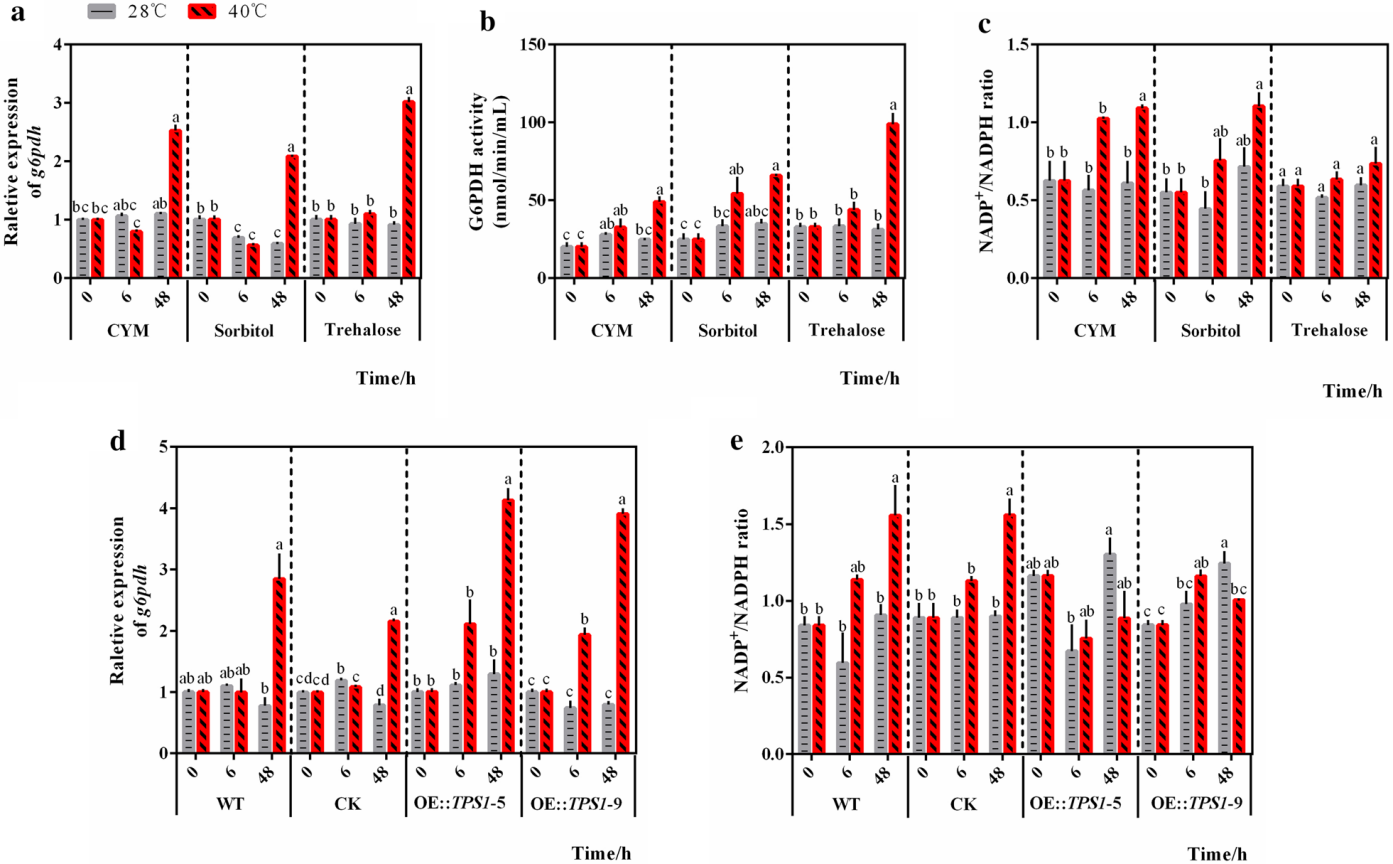
Trehalose can enhance PPP activity in *P. ostreatus* under heat stress. The WT strains were transferred to new CYM medium with or without trehalose or sorbitol after 5 days of culture and treated with heat (40 °C). Mycelia were then collected for determination of **a** the relative gene expression level of *g6pdh*, **b** the enzymatic activity of G6PDH, and **c** the NADP^+^/NADPH ratio. The WT strain, CK strain, and *tps*1 overexpression strains were treated with heat (40 °C) after culture at 28 °C for 5 days and then collected for determination of **d** the relative gene expression level of *g6pdh* and **e** the NADP^+^/NADPH ratio. n = 3; the data are expressed as mean ± SEM values. The significance analysis was conducted within CYM, Sorbitol, or Trehalose group; within WT, CK, OE::TPS1-5, or OE::TPS1-9 group separately, and the significant differences were marked with different lowercase letters over the columns (*P* < 0.05 according to the Tukey or Kruskal-Wallis test), the lowercase letters among different groups have no comparability in significance analysis

To clarify the mechanism by which *hk* expression increases under heat stress in the presence of trehalose, the gene transcription levels and enzymatic activity of G6PDH, the key enzyme in the PPP, were determined. Compared with the corresponding levels in the CYM control and sorbitol control groups, *g6pdh* gene expression was significantly increased (2.53 ± 0.08 vs. 3.02 ± 0.06, *P* = 0.010; 2.09 ± 0.01 vs. 3.02 ± 0.06, *P* < 0.001) in the group treated with trehalose and subjected to heat stress for 48 h (Fig. 3a). Treatment with exogenous trehalose for 48 h under heat stress also significantly increased G6PDH enzyme activity compared with the CYM (49.02 ± 2.93 vs. 98.91 ± 6.39, *P* = 0.002) and sorbitol (65.91 ± 0.46 vs. 98.91 ± 6.39, *P* = 0.007) controls (Fig. 3b). These results collectively indicated that exogenous trehalose could further enhance PPP activity under heat stress. The level of NADPH, an important product of the PPP, increased significantly (0.76 ± 0.06 vs. 0.61 ± 0.02, *P* = 0.025) in the presence of trehalose under heat stress compared with the CYM control (Fig. 3c), which again verified the promotive effect of trehalose on the PPP under heat stress. We evaluated the PPP in the transformants OE::TPS1-5 and OE::TPS1-9 and found that the *g6pdh* gene expression level was indeed significantly higher (2.85 ± 0.40 vs. 4.13 ± 0.18, *P* = 0.042; 2.85 ± 0.40 vs. 3.91 ± 0.07, *P* = 0.046) (Fig. 3d) than the WT group after heat stress for 48 h. High temperature treatment for 48 h had different effects on the NADP^+^/NADPH ratio in each group (Fig. 3e). The NADP^+^/NADPH ratio was increased in WT and CK groups, and was decreased in OE::TPS1-5 and OE::TPS1-9 groups. The results further showed the increase of the NADPH content in the transformants compared with the control groups after heat stress. These results indicated the effect of trehalose on enhancing PPP activity in *P. ostreatus* under heat stress and explained why trehalose downregulated the expression of key glycolytic enzymes except *hk* under heat stress.

**Fig. 4:**
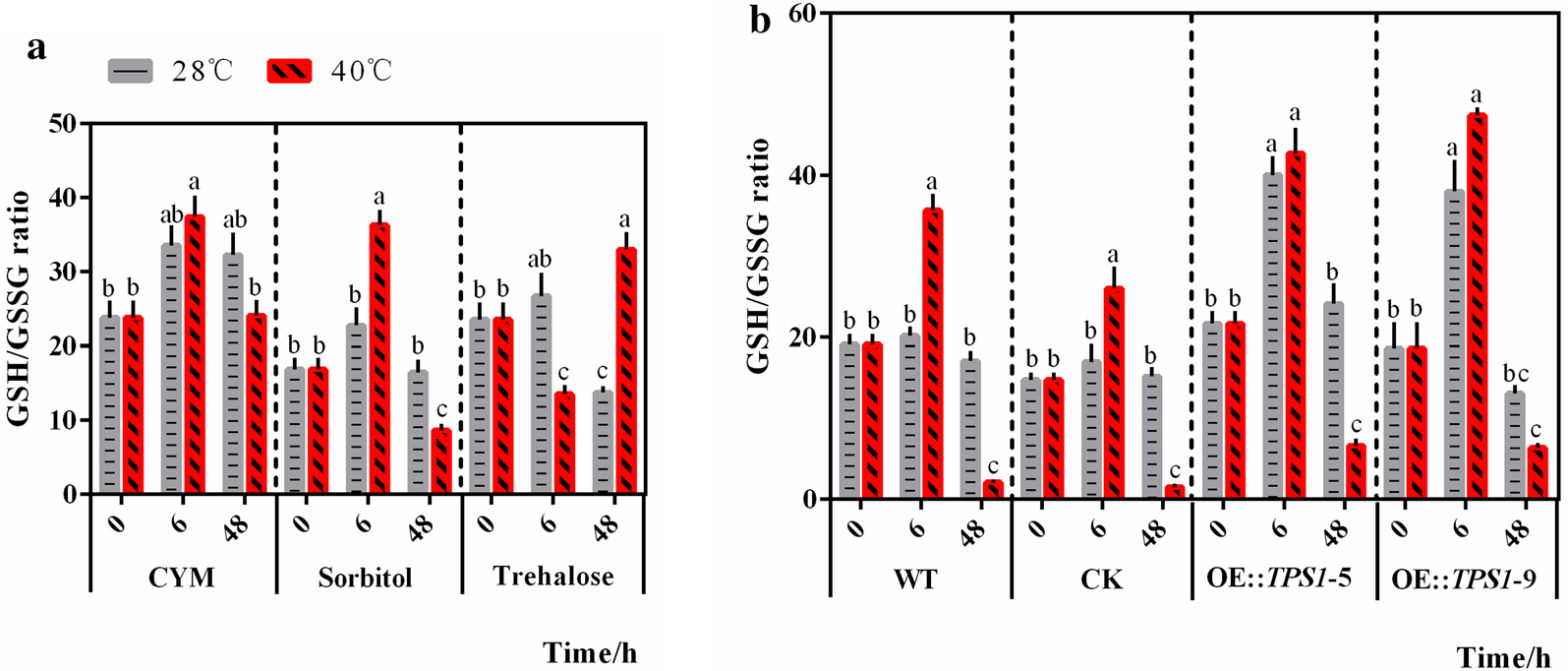
Trehalose alleviates heat stress by enhancing PPP activity in *P. ostreatus*. **a** The WT strains were transferred to new CYM medium with or without trehalose or sorbitol after 5 days of culture and treated with heat (40 °C), and **b** the WT strain, CK strain, and *tps*1 overexpression strains were treated with heat (40 °C) after culture at 28 °C for 5 days. Then, samples of the strains were collected for determination of the GSH/GSSG ratio. n = 3; data are expressed as mean ± SEM values. The significance analysis was conducted within CYM, Sorbitol, or Trehalose group; within WT, CK, OE::TPS1-5, or OE::TPS1-9 group separately, and the significant differences were marked with different lowercase letters over the columns (*P* < 0.05 according to the Tukey or Kruskal–Wallis test), the lowercase letters among different groups have no comparability in significance analysis

An increase in NADPH helps to ameliorate the oxidative environment in cells; thus, we also evaluated the intracellular redox pair GSH/GSSG to assess the redox state in cells [22]. As shown in Fig. 4a, comparing the GSH/GSSG ratio at 28 °C and 40 °C in each group, we found that the GSH/GSSG ratio in the group treated with trehalose after 48 h of high-temperature stress was greatly increased compared with the CYM control and sorbitol control groups. Even the GSH/GSSG ratio decreased in the group treated with trehalose after 6 h of high temperature stress for unknown reasons, and finally an increase in GSH production was observed in the long-term processing time with trehalose. However, in the transformants OE::TPS1-5 and OE::TPS1-9 (Fig. 4b), the GSH/GSSG ratio did not increase after 48 h of high-temperature stress compared with the exogenous trehalose groups. When compared with the WT and CK groups, the GSH/GSSG ratio in OE::TPS1-9 treated with 48 h of high temperature, and in both OE::TPS1-5 and OE::TPS1-9 treated with 6 h of high temperature showed the changes, which indicated the positive effect of *tps*1 overexpression on GSH production. The effect of *tps*1 overexpression on GSH content may less than that of exogenous trehalose. In addition, the content of GSH may be affected by many complex factors, such as Ca^2+^ [23], melatonin [24], spermidine [25], γ-aminobutyrate (GABA) [26] and so on. Overall, both treatment with exogenous trehalose (Fig. 4a) and overexpression of *tps*1 (Fig. 4b) tended to promote GSH production under heat stress, thus probably enhance the elimination of active oxygen radicals. Based on previous studies in our laboratory, which relay the effect of trehalose on oxidative damage in *P. ostreatus* under heat stress [16], we can draw a preliminary conclusion that trehalose can alleviate oxidative damage in *P. ostreatus* under heat stress by enhancing PPP activity.

### **Trehalose can alleviate the decrease in the oxygen consumption rate caused by heat stress**

**Fig. 5:**
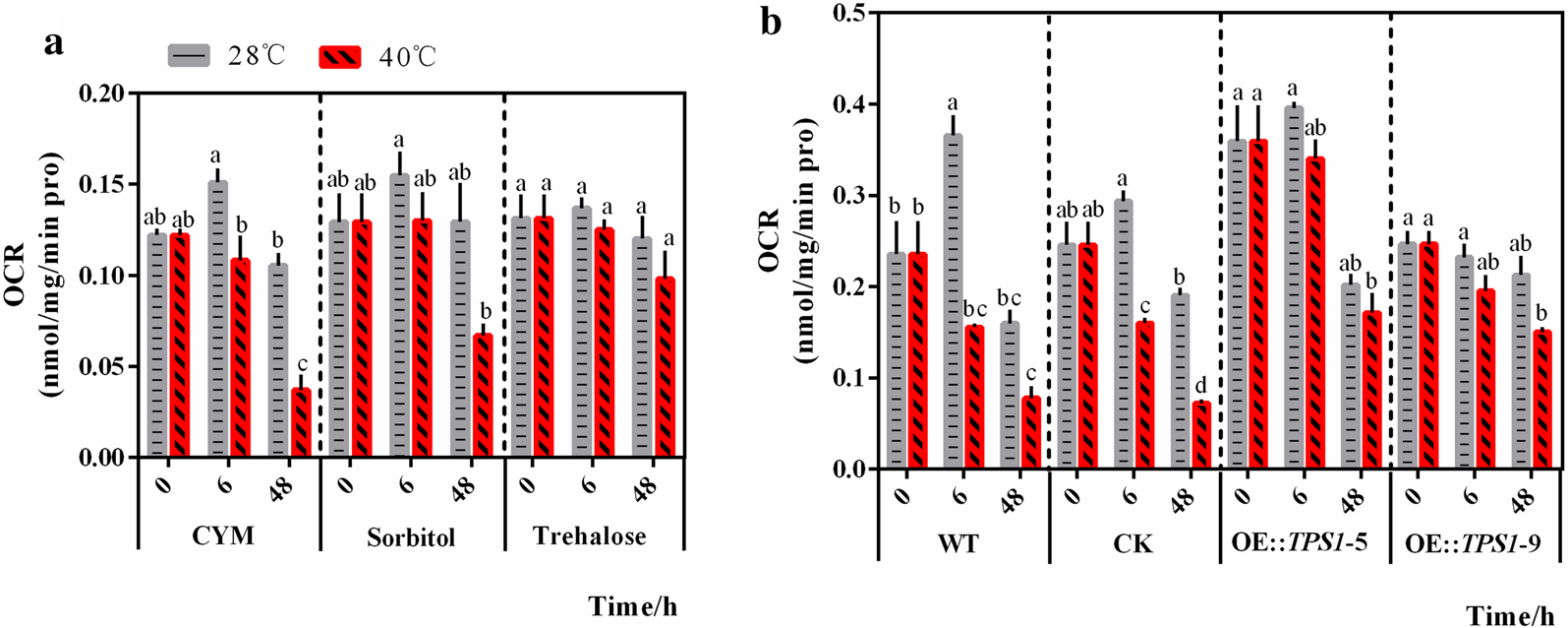
Trehalose can alleviate the decrease in the oxygen consumption rate caused by heat stress. **a** The WT strains were transferred to new CYM medium with or without trehalose or sorbitol after 5 d of culture and treated with heat (40 °C), and **b** the WT strain, CK strain, and *tps*1 overexpression strains were treated with heat (40 °C) after culture at 28 °C for 5 d. Then, samples of the strains were collected for measurement of the oxygen respiration rate (OCR). n = 3; data are expressed as mean ± SEM values. Pro: protein. The significance analysis was conducted within CYM, Sorbitol, or Trehalose group; within WT, CK, OE::TPS1-5, or OE::TPS1-9 group separately, and the significant differences were marked with different lowercase letters over the columns (*P* < 0.05 according to the Tukey or Kruskal–Wallis test), the lowercase letters among different groups have no comparability in significance analysis

The above results showed the effect of trehalose on alleviating heat stress by inhibiting glycolysis and enhancing PPP activity. The regulatory role of trehalose in these central carbon metabolism pathways stimulated our interest in its effect on mitochondrial function. We found that the decrease in the OCR of *P. ostreatus* under heat stress in CYM groups can be alleviated by trehalose addition (Fig. 5a). Compared to the WT and CK strains, the transformants OE::TPS1-5 and OE::TPS1-9 exhibited a good recovery effect on the OCR decrease at 6 h (40 °C–6 h with 28 °C–6 h in each group), but not at 48 h (Fig. 5b). These results revealed the effect of trehalose on improving oxygen respiration in mycelia under high-temperature stress. Cellular respiration is a useful criterion for evaluating mitochondrial dysfunction [27]; thus, a certain relationship might exist between trehalose and mitochondrial function under high-temperature stress.

## Discussion

In this study, we measured the expression levels of genes encoding key enzymes and the content of key metabolites in glycolysis and the PPP under heat stress in *P. ostreatus* strains treated with exogenous trehalose or *tps*1 overexpression. We also evaluated the effect of trehalose on the OCR in *P. ostreatus* mycelia. Our results showed that trehalose could alleviate heat stress in *P. ostreatus* by inhibiting glycolysis and enhancing PPP activity, indicating the regulatory effect of trehalose on central carbon metabolism in defense against high-temperature stress in *P. ostreatus*.

The regulatory effect of trehalose on metabolism during stress relief is not unusual. The trehalose pathway can alleviate glucose stress by regulating glycolysis in *S. cerevisiae* [28]. In addition, trehalose regulates the PPP in pathogenic fungi during infection [29]. Moreover, trehalose mediates thermotolerance in *Gluconobacter frateurii*; research has shown that trehalose does not contribute to thermotolerance directly but that a metabolic change including increased carbon flux to the PPP is instead the key factor [30]. However, the studies above reflect the role of trehalose in a single metabolic pathway, and few reports have addressed the regulatory effects of trehalose in multiple metabolic pathways and in redirecting metabolic flux. Considering the important role of metabolic transformation in stress defense [31, 32], our results further illustrate the importance of trehalose in stress defense.

Among the altered central carbon metabolism pathways, the PPP is considered an alternative pathway for the oxidative decomposition of glucose. Because the PPP can provide a variety of raw materials for anabolism, PPP activity was once thought to be enhanced only in cells with increased cellular anabolism. However, the key product of PPP is NADPH. Because NADPH is a central cofactor in the GSH and thioredoxin/peroxiredoxin antioxidant systems, an increase in NADPH increases the production of GSH, thus scavenging much greater amounts of ROS and contributing to ameliorating the oxidative cellular environment [33]. Thus, the PPP can be considered to represent the stress resistance ability. In our study, PPP activity was enhanced under high-temperature stress, and this increase became more obvious after trehalose addition, demonstrating the function of PPP in *P. ostreatus* stress defense. Regarding the mechanism by which trehalose enhances PPP activity, some studies suggest that substrate competition occurs between trehalose biosynthesis and the PPP, considering G6P consumption [34]. Other studies suggest that this enhancing effect may be driven by *tps*1. *Tps*1, a gene encoding a key enzyme in trehalose metabolism, can directly activate G6PDH by sensing G-6-P to produce NADPH via the PPP [35].

The intermediate metabolites of glycolysis can participate in diverse biosynthetic pathways and help cells grow and divide rapidly. In addition, glycolysis produces small amounts of ATP and NADH, and its end product (pyruvate) is the direct source of lactic acid in tumor cells [21]. Regarding the increase in glycolysis under stress, in addition to our study of high-temperature stress, another study reported such an increase under chromium (Cr) (VI) stress. Cr (VI) induces aerobic glycolysis in A549 cells (a human lung carcinoma epithelial cell line), and glycolysis is considered to increase the intracellular redox potential and to attenuate cell death and apoptosis [36]. However, the significance of glycolytic induction under stress still requires further exploration. Considering the accumulation of lactic acid, which can inhibit the mycelial growth of *P. ostreatus* [20], the increase in glycolysis under high-temperature stress is unfavorable to *P. ostreatus*. Therefore, the effect of trehalose on alleviating the increase in glycolysis is a reflection of the ability of trehalose to relieve high-temperature stress. Early reports have implicated the trehalose biosynthetic pathway in the control of glycolysis in yeast [37]. Exogenous trehalose was shown to inhibit glucose transport efficiency, and the increased intracellular trehalose content destroyed the equilibrium of the trehalose cycle and caused glycolytic instability [38]. One group believed that the product of the *tps*1 gene restricts the influx of glucose into glycolysis, thus controlling glycolytic pathway flux in *S. cerevisiae* [39]. Others have attributed this effect to *tps*1 and reported that *tps*1 is a sugar sensor that senses G-6-P to regulate glucose metabolism in *Magnaporthe oryzae* [40] and carbon metabolism in *S. cerevisiae* [41]. Moreover, studies have shown that the Δ*tps*1 strain indeed has higher metabolic flux through the glycolytic pathway [42, 43]. Perhaps *tps*1 plays a more dominant role than trehalose in the defense against high-temperature stress; this possibility requires more comprehensive research.

We found that trehalose can alleviate the decrease in the OCR of *P. ostreatus* under heat stress. Mitochondria are the main site of cellular aerobic respiration. Therefore, the OCR is an indicator of mitochondrial function. This result may suggest a relationship between trehalose and mitochondrial function. As previous studies have reported, trehalose can ameliorate oxidative stress-mediated mitochondrial dysfunction [17], and the trehalose pathway can regulate mitochondrial respiratory chain content in *S. cerevisiae*. Additionally, the respiratory rate in the Δ*tps*1 strain reduced significantly compared with wild type [39], suggesting a role for trehalose in regulating OCR and oxidative phosphorylation and the involvement of *tps*1 in the regulation of mitochondrial biogenesis. In addition, the Δ*tps*1 mutant showed an enhanced oxidative phosphorylation capacity [42]. However, mitochondria can also regulate trehalose metabolism; for example, the presence of rotenone prevents trehalose accumulation, suggesting a potential relationship between the synthesis of trehalose and the electron transport chain [44]. As the regulatory center of metabolism, mitochondria may play a more important role than we know in stress defense. We speculate that the effect of trehalose on the OCR may affect mitochondrial function and thus promote resistance to heat stress.

Because of the functional diversity of trehalose, its application in heat stress defense requires more comprehensive research. The function of *tps*1 in mycelia of edible fungi also requires further clarification.

## Conclusions

**Fig. 6:**
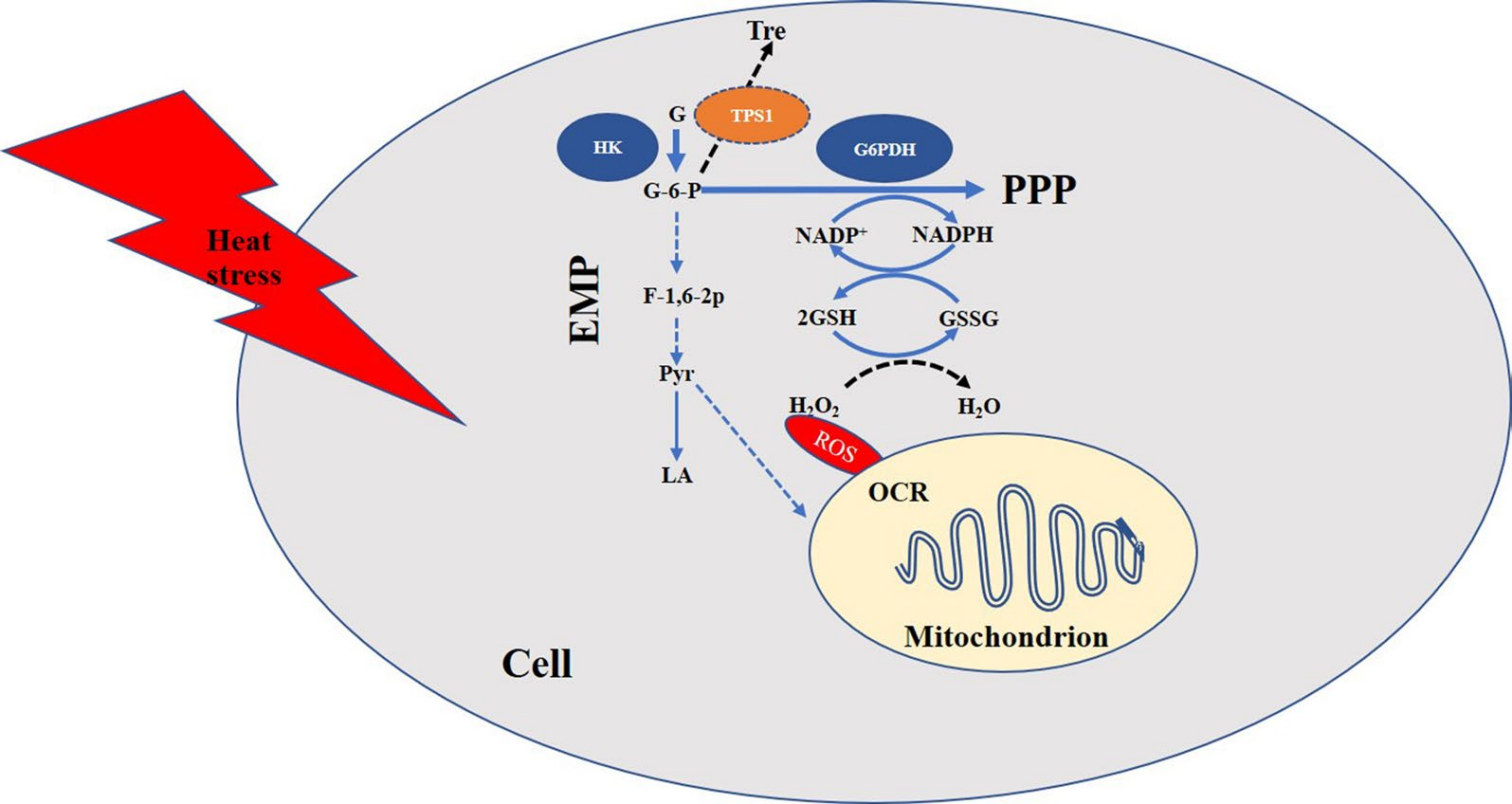
Schematic illustrating the ameliorative effect of trehalose on heat stress in *P. ostreatus*. Trehalose decreased the content of lactate and downregulated the expression of the key glycolytic enzymes, except *hk*, thus alleviating the increase in glycolysis in *P. ostreatus* under heat stress. In addition, trehalose increased the gene expression and enzymatic activity of G6PDH, enhanced PPP activity, and increased NADPH production. Thus, the conversion of GSSG into GSH was increased by the conversion between NADPH and NADP^+^, and greater amounts of ROS would be scavenged in cells. Moreover, trehalose alleviated the decrease in the OCR caused by heat stress. Tre: trehalose, G: glucose, HK: hexokinase, G-6-P: glucose 6-phosphate, F-1,6-2P: fructose-1,6−2 phosphate, Pyr: pyruvate, LA: lactate, EMP: glycolysis, TPS1: trehalose-6-phosphate synthase 1, G6PDH: glucose-6-phosphate dehydrogenase, PPP: pentose phosphate pathway, OCR: oxygen consumption rate. The black dashed line indicates results from other reports, the blue dashed lines indicate the omitted intermediate steps, and the blue solid lines indicate results obtained in this study

The results of this study suggest that both treatment with exogenous trehalose and endogenous overexpression of *tps*1 can alleviate heat stress (as shown in Fig. 6). Trehalose can alleviate heat stress by suppressing the increases in glycolysis and lactate accumulation under heat stress. It can also enhance PPP activity and increase NADPH production by enhancing the transcription and enzymatic activity of G6PDH, and further increase the GSH content to alleviate heat stress together. Moreover, trehalose can suppress the decrease in the OCR under heat stress by regulating some undetermined pathways related to mitochondria, which may also help to alleviate heat stress. The role of trehalose in mechanism regulation provides a new research direction and basis for physiological study on the stress resistance of edible fungi.

## Methods

### Strains and culture conditions

The strain used in this study was *P. ostreatus* 389 (CCMSSC 00389, dikaryon), which was obtained from the China Center for Mushroom Spawn Standards and Control.

For the exogenous trehalose addition experiments described in this paper, the wild-type (WT) strains were cultured at 28 °C on CYM medium for 5 days and then transferred to new CYM medium with or without 1.5% (w/v) trehalose or 1.5% (w/v) sorbitol according to a previous study [2], after which they were treated with heat (40 °C) for 0, 6, and 48 h. The composition of the CYM medium was 2% glucose, 1% maltose, 0.05% MgSO_4_·7H_2_O, 0.2% yeast extract, 0.46% KH_2_PO_4_, and 0.2% tryptone [45].

For the endogenous trehalose overexpression experiments described in this paper, TPS1 overexpression strains generated previously by our lab were used [2]. Tps1 catalyzes the first step in trehalose synthesis, and the *tps*1 expression levels in transformants OE::TPS1-5 and OE::TPS1-9 were up-regulated 2.44 and 2.56 times compared with WT, respectively. The intracellular trehalose content in OE::TPS1-5 and OE::TPS1-9 were increased to 99.94 mg/g fresh weight (FW) and 105.67 mg/g FW at 28 °C and to 176.28 mg/g FW and 175.99 mg/g FW at 40 °C (the trehalose content was approximately 72.85 mg/g FW in WT at 28 °C and 153.16 mg/g FW in WT at 40 °C) [2, 46]. The WT strain, CK strain (control transformed with the empty vector), and TPS1 overexpression strains were cultured at 28 °C for 5 d on CYM medium and then treated with heat (40 °C) for 0, 6, and 48 h.

Metabolomic analysis of *P. ostreatus* mycelia by liquid chromatography-mass spectrometry (LC–MS)

Metabolomic analysis was performed on *P. ostreatus* mycelia treated with heat (40 °C) for 0–48 h by LC–MS according to previously described methods [47]. All treatments included controls incubated at 28 °C.

### Lactate assay

Lactate was assayed by ultrahigh-performance liquid chromatography (UPLC) according to a previous study [20].

### RNA extraction, reverse transcription, and real-time PCR analysis of gene expression

Total RNA was extracted using a FastPure Plant Total RNA Isolation Kit (Polysaccharides & Polyphenolics-rich) (Vazyme Biotechnology, Nanjing, China), and cDNA was synthesized using a HiScript® II 1st Strand cDNA Synthesis Kit (+gDNA Wiper) (Vazyme Biotechnology) according to the manufacturer’s instructions. The mRNA levels of different genes were measured by quantitative real-time PCR with ChamQ Universal SYBR qPCR Master Mix (Vazyme Biotechnology). The sequences of the primers used are listed in Additional file 1: Table S1. Gene expression was evaluated by calculating the differences between the Ct values of the target genes and those of the GAPDH gene (reference gene).

### Enzyme activity assay

Glucose-6-phosphate dehydrogenase (G6PDH) enzyme activity was assessed with a Glucose-6-Phosphate Dehydrogenase Assay Kit (Sigma-Aldrich, Merck, Darmstadt, Germany) according to the manufacturer’s instructions.

### Oxidized/reduced nicotinamide‐adenine dinucleotide phosphate (NADP^+^/NADPH) assay

The NADP^+^/NADPH ratio was determined with a CheKine™ NADP/NADPH Assay Kit (Abbkine Scientific, Wuhan, China) according to the manufacturer’s instructions.

### Oxidized glutathione/glutathione (GSSG/GSH) assay

The GSSG/GSH ratio was determined with a GSSG/GSH Quantification Kit II (Dojindo Laboratories, Kumamoto, Japan) according to the manufacturer’s instructions.

### Measurement of oxygen consumption rates (OCRs)

OCRs were measured using a Hansatech Oxy-lab (Hansatech Instruments, Norfolk, UK) according to a previous method with modifications [48]. Homogenized mycelial pellets cultured in liquid medium were used for measurement. A 0.5 mL volume of mycelial pellets from each treatment group was added to the reaction chamber for OCR measurement, and another 0.5 mL of sample was collected for the corresponding protein content assay.

### Statistical analysis

For the metabolomic assays, seven biological repeats were performed, and the data are presented as the mean normalized values referring to previous literature [49]. For the other assays, at least three biological replicates were performed, and the data are presented as mean ± SEM values. Statistical significance was defined as *P* < 0.05. All statistical analyses were performed using GraphPad Prism 6 (GraphPad Software Inc., San Diego, CA, USA), SPSS 20.0 software (SPSS Inc., Chicago, IL, USA), and Excel 2010 software (Microsoft, Redmond, WA, USA).

## Supplementary Information

**Additional file 1.** Primer sequences.

## Data Availability

All data generated or analyzed during this study are included in this published article.
