# Supplementary material for: Trehalose alleviates high‐temperature stress in Pleurotus ostreatus by affecting central carbon metabolism
Source: Microb Cell Fact. 2021 Apr 7;20:82. doi: 10.1186/s12934-021-01572-9 (PMC8028756; doi:10.1186/s12934-021-01572-9)
Supplement: Supplementary file 1 — Additional file 1. Primer sequences. [file 12934_2021_1572_MOESM1_ESM.pdf]

Additional file 1: Table S1 Primer sequences

Table S1 Primer sequences

Name	Oligonucleotide sequence (5’ to 3’)
HK F	CATCGACCGACATTTCCA
HK R	TTCAGTACCACCTCGCAGA
PFK F	TTGGGAAGACGGAATGTG
PFK R	TCAAGTCCTAGCCGCTCA
PK F	CTGAACAACGGAACCCTC
PK R	GGATGAATGACGCAAAGA
G6PDH F	GTGACATCCTCCAGAACCA
G6PDH R	GCCATTTGCGGCGACATA
GAPDH F	GTGTTAACCTCGAGACTTACG
GAPDH R	TGGTGGCGTGGATTGTGCTC
